# The development and validation of a new interprofessional team approach evaluation scale

**DOI:** 10.1371/journal.pone.0201385

**Published:** 2018-08-09

**Authors:** Zalika Klemenc-Ketis, Irena Makivić, Antonija Poplas Susic

**Affiliations:** 1 Community Health Centre Ljubljana, Ljubljana, Slovenia; 2 Department of Family Medicine, Faculty of Medicine, University of Ljubljana, Ljubljana, Slovenia; 3 Department of Family Medicine, Faculty of Medicine, University of Maribor, Maribor, Slovenia; University of North Carolina at Chapel Hill, UNITED STATES

## Abstract

A team approach in health care involves an interprofessional approach to patient care. We wanted to develop and validate a tool that would evaluate the interprofessional team approach to patients of a family medicine team. We performed a descriptive study in three consecutive phases: a literature review, consensus development panels, and a cross-sectional validation study. Three rounds of consensus development panels were carried out in order to evaluate and adapt the initial scale. The cross-sectional study was carried out in all Slovenian family medicine practices, each invited 10 consecutive patients. In the quantitative study, 3,292 patients participated (a 50.7% response rate), of which 1,810 (55.0%) were women. The mean age of the sample was 53.1 ± 1.2 years. The final Cronbach’s alpha was 0.901. A factor analysis of the 9-item scale put forward two factors (Team Approach and Person-Centred approach) which explained 68.6% of the variance. This study provided a new scale for the evaluation of patient satisfaction with the interprofessional family medicine team from the patients’ point of view. It opened the question of family medicine team competencies and pointed towards the need to develop a family medicine interprofessional team competency framework and a comprehensive tool for its assessment.

## Introduction

Person-centred care is one of the main competencies of family medicine [1] and encompasses the ability to provide longitudinal continuity of care as determined by the needs of the patient, referring to continuing and coordinated care management [2]. Person-centred care requires interdisciplinary and interprofessional teamwork in order to enable the management of the patient as a whole person [3]. Teamwork is defined as the provision of health services to individuals, families, and/or their communities by at least two health providers who work collaboratively with patients and their caregivers—to the extent preferred by each patient—to accomplish shared goals within and across settings to achieve coordinated, high-quality care [4]. Interdisciplinary team work is a complex process in which different types of staff work together to share expertise, knowledge, and skills to impact on patient care [5].

A team approach involves an interprofessional and coordinated approach to patient care, and requires clear definitions of the individual team members’ roles. A team approach to patients in family medicine has been shown to achieve better outcomes, a higher quality of care, worker satisfaction, and cost containment [6].

The theoretical framework that describes an interprofessional team-based primary care includes four domains: cognitive domain, affective/relational domain, behavioural domain, and leadership. There are almost 50 instruments that measure interprofessional team-based primary care according to the mentioned framework. Mostly, these are surveys, focusing on team member, and include at least three of the framework’s domains [7].

In 2011, an ongoing project at the primary care level in Slovenia was launched, with the support of the Ministry of Health. It introduced a new model of family medicine practice, where a family physicians’ working team, which at that time consisted of a family physician and a practice nurse, was extended by a nurse practitioner, with very well-defined competencies, working four hours per day or 0.5 full-time equivalents [8]. This approach implemented an interprofessional team practising person-centred care in Slovenian family medicine [8–12]. As a new team member was added to the family medicine practice team within the project, we developed a tool to measure patient satisfaction with the nurse practitioners, which evaluates their clinical approach, comprehensive approach, and patient-centred approach [9].

The limitation of the tools used to evaluate patient satisfaction in the Slovenian family medicine practices involved in the project was that they evaluated the experience of patients with one member of the team, not with the team as a whole. This was not in line with the interprofessional team-based nature of this project.

We therefore decided to develop a tool which would assess the interprofessional family medicine team approach from the patients’ viewpoint. This approach to patients is based on a theoretical framework of the patient management in Slovenian family medicine practices described elsewhere [13].

## Materials and methods

### Research design

**Fig 1:**
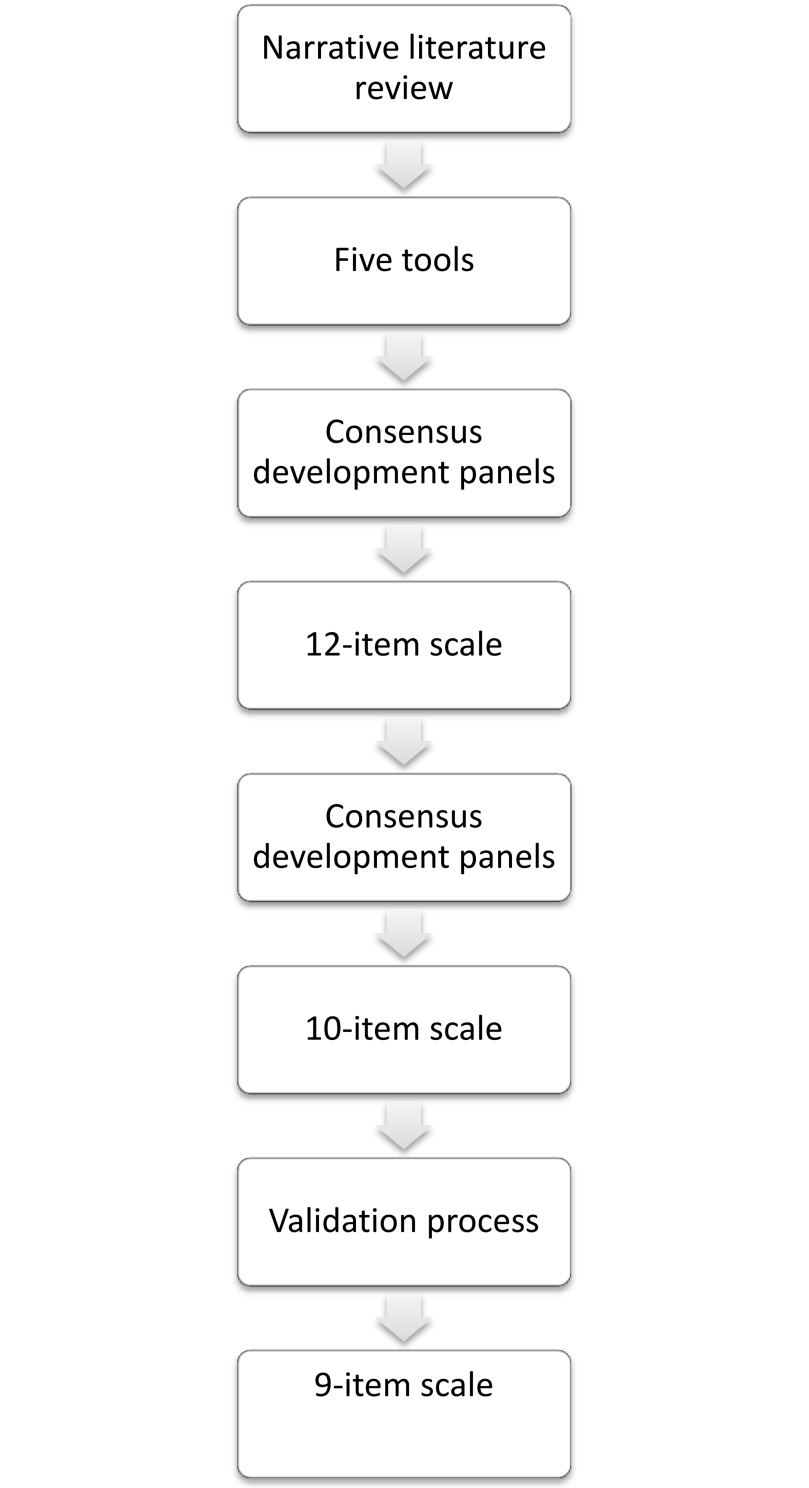
Flow-chart of the scale development. This flow-chart describes a step-wise development of the new interprofessional team approach scale.

We carried out a descriptive study in three consecutive phases: a literature review, consensus development panels, and a cross-sectional validation study (Fig 1). Each subsequent phase was informed by and built on the preceding phase.

### Data collection

We carried out a narrative literature review in order to identify existing tools for the evaluation of the team approach to patients in primary health care. We used the following key words: Teamwork; Team Work; Interprofessional Team-based Approach; Family Practice; Family Medicine; Primary Health Care; Primary Healthcare; Patient Satisfaction. We searched in the following databases: MEDLINE (1966–2016), ISI Web of Science (1970–2016), ProQuest (1980–2016), EMBASE (1980–2016), SCOPUS (1960–2016), Springer Link (1980–2016), Sage Journals (1980–2016) and CINAHL (1982–2016) (Box 1).

**Box 1. Full electronic search strategy used in the narrative review**

#1. Teamwork/Team Work
#2. Interprofessional Team-Based Approach
#3. Family Practice/Family Medicine
#4. Primary Health Care/Primary Healthcare
#5. Patient Satisfaction
#6. (#1 OR #2 OR #5) IN #3 OR #4

Two researchers independently reviewed the scales identified through the process described above and prepared an initial scale, with items based on the identified scales. The items were translated to Slovene language by a standard forward-backwards translation procedure [14].

Two rounds of consensus development panels were carried out in order to evaluate and adapt the initial scale. Experts from family medicine (N = 4) and nursing (N = 4) participated in these panels. These experts were very familiar with the theoretical framework of the patient management in Slovenian family medicine practices [13]. Both panels took place in face-to-face meetings in 2016. During the first meeting, the initial scale was reviewed, and the experts gave their opinions and suggestions. During the second meeting, the final content of the scale in Slovene language was approved.

A cross-sectional study was carried out in January 2017 in Slovenian family medicine practices. All the family medicine practices in Slovenia that had implemented the new approach to patients [13] described in the introduction were invited to participate (N = 649). Each family medicine practice invited 10 consecutive patients who attended the practice on the same day to participate in the study. Patients that were unable to fill in the questionnaire (e.g. due to blindness, cognitive impairment, or terminal illness) or who did not want to participate or were younger than 18 years were excluded from the study. One member of the practice team explained to the patients the aim and method of the study and invited them to participate. They gave an oral informed consent. They filled-in the questionnaire in the waiting room and put it in the sealed box afterwards to secure the confidentiality of the data.

The questionnaire consisted of the newly developed 10-item Interprofessional Team Approach Evaluation Scale, and of questions on the demographic characteristics of the patients. The items could be answered on a 5-point Likert scale expressing the level of agreement with the items (1 –I don’t agree at all; 5 –I totally agree).

### Data analysis

The data were analysed by SPSS 25.0 (SPSS Inc. Chicago, IL, USA). The face validity of the scale was confirmed by the consensus development panels described above. The content validity was determined through consensus development panels where the experts judged the appropriateness of the scale to measure all aspects if the underlying construct. The construct validity of the questionnaire was determined by factor analysis. Prior to that, we determined the multicollinearity and calculated the Kaiser-Meyer-Olkin (KMO) measure of sampling adequacy and Bartlett’s test of sphericity. Exploratory factor analysis was performed, and a rotation method employed was Oblimin with Kaiser normalisation. Items with communalities less than 0.3 were excluded from the final scale [15,16]. The reliability of the scale was determined by Cronbach’s alpha.

Due to the high Cronbach’s alpha value, we calculated a composite score. We used a Baker & Hearnshaw equation [17] [(Σitems 1–i) × 100/(5 × i)] × 1.25–25) to range the scale’s score from 0 to 100. In this equation, Σitems 1–i represents the sum of the scores of i items; 5 represents the maximum score points of each item; and i represents the number of items. The other numbers are needed for mathematical purposes in order to range the scale’s score from 0 to 100.

For each factor, we also calculated the Cronbach’s alpha.

To test the normality of the dataset, we used the Shapiro-Wilk test.

### Ethical consideration

The study received ethical approval from the National Ethics Committee of the Republic of Slovenia (No. 79/06/12).

## Results

### Literature review

Through the narrative literature review, we identified five tools that could be used to measure patient evaluation of the team approach in primary health care settings: the General Practice Assessment Questionnaire [18], the Primary Care Assessment Tool [19], the Nurse Practitioners Satisfaction Survey [20], the General Practice Nurse Satisfaction Scale [21], and the Consultation Satisfaction Questionnaire [22].

### Consensus development panels

An initial 12-item scale was developed by the experts after the first consensus development panel. After the second consensus development panel, two items were removed and all the other ten items were reformulated. The 10-item scale was presented to the third consensus development panel and was accepted by the experts after minimal modifications in wording.

### Cross-sectional validation study

A total of 3,292 patients participated in this study (a 50.7% response rate), of which 1,810 (55.7%) were women (Table 1). The mean age ± standard deviation of the sample was 53.1 ± 1.2 years.

**Table 1:** Demographic characteristics of the sample.

Characteristic	Number (%)
Gender	
Male	1439 (43.3)
Female	1810 (55.7)
Registered by	
Public family physician	2289 (69.6)
Family physician with concession	1002 (30.4)
Region of Slovenia	
Celje	415 (12.6)
Koper	193 (5.9)
Krško	141 (4.3)
Kranj	191 (5.8)
Ljubljana	939 (28.5)
Maribor	422 (12.8)
Murska Sobota	280 (8.5)
Nova Gorica	267 (8.1)
Novo Mesto	219 (6.7)
Ravne na Koroskem	224 (6.8)
Education	
Unfinished primary school	69 (2.1)
Primary school	512 (15.8)
Vocational school	804 (24.8)
Secondary school	1001 (30.9)
University, PhD	855 (26.4)
Employment status	
Employed	1804 (56.1)
Unemployed	350 (10.9)
In school	47 (1.5)
Retired	1013 (31.5)
Chronic disease	
Present	1512 (48.8)
Not present	1586 (51.2)

The Kaiser-Meyer-Olkin (KMO) measure of sampling adequacy was 0.918 and its significance (Bartlett’s test of sphericity) was < 0.001. The communalities of items were in general higher than 0.3, except for item 1 (Table 2).

**Table 2:** Items’ communalities (extraction method: principal axis factoring).

Item No.	Item	Initial	Extraction
1	When making an appointment in this practice, I had to give the reason for an appointment.	0.033	0.030
2	The appointment system in this practice is flexible.	0.339	0.362
3	During the consultation, team members gave me enough information on self-care.	0.562	0.684
4	During the consultation, team members gave me understandable information about my health and planned treatment.	0.592	0.679
5	During the consultation, I was able to express my expectations regarding my treatment plan to the team.	0.504	0.567
6	In this practice, all patients are treated equally by the whole team.	0.517	0.553
7	Each team member in this practice knows their role.	0.574	0.633
8	Team members in this practice respect each other.	0.592	0.676
9	In this practice, the team handles my data confidentially.	0.550	0.616
10	In this practice, all patients are treated with respect by the whole team.	0.581	0.638

The rotated solution extracted two factors. Item 1 was removed as its value was < 0.3 (Table 3). So, the scale was left with 9 items. Two factors had initial eigenvalues more than one. This factor analysis explained 54.4% of variance (Factor 1 explained 48.1% and Factor 2 6.3% of the variance).

**Table 3:** Factor analysis of the 9-item interprofessional team approach evaluation scale.

Item No.	Item	Factor 1 –Team Approach	Factor 2 –Person-Centeredness
1	When making an appointment in this practice, I had to give the reason for an appointment.	Removed	removed
2	The appointment system in this practice is flexible.		0.523
3	During the consultation, team members gave me enough information on self-care.		0.872
4	During the consultation, team members gave me understandable information about my health and planned treatment.		0.809
5	During the consultation, I was able to express my expectations regarding my treatment plan to the team.		0.662
6	In this practice, all patients are treated equally by the whole team.	0.627	
7	Each team member in this practice knows their role.	0.761	
8	Team members in this practice respect each other.	0.879	
9	In this practice, the team handles my data confidentially.	0.802	
10	In this practice, all patients are treated with respect by the whole team.	0.770	

**Fig 2:**
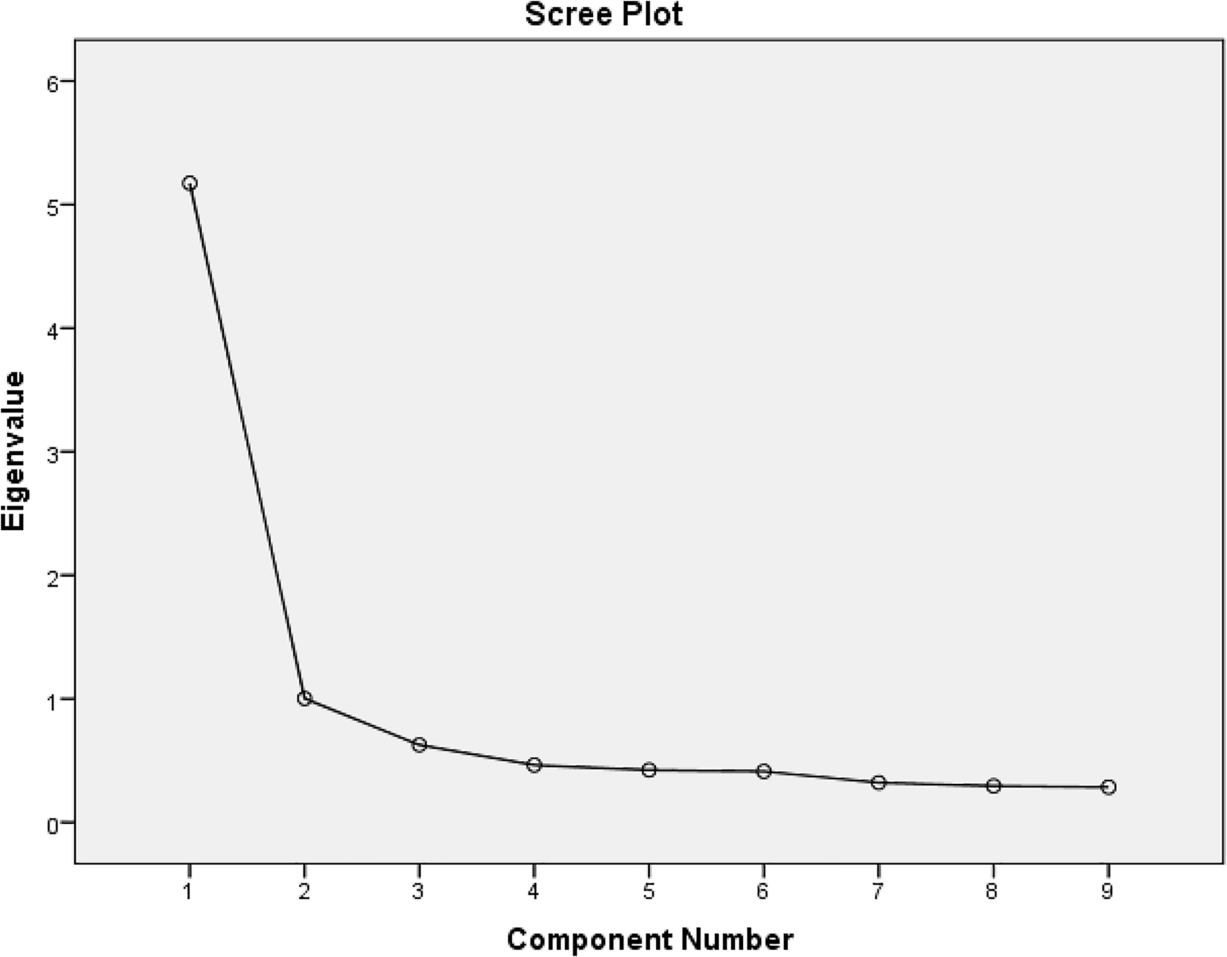
The scree plot of a 10-item interprofessional team approach evaluation scale. This scree plot shows the variance explained by each factor in a factor analysis.

The scree plot showed that more variance could be explained by two factors than only one (Fig 2).

The reliability analysis of the 10-item scale showed that the Cronbach’s alpha was 0.774. When item 1 was removed, the value of the Cronbach’s alpha increased to 0.901 (Table 4). The Cronbach’s alpha of factor 1 was 0.827 and of factor 2 was 0.886.

**Table 4:** Interprofessional team approach evaluation scale: 10- and 9-item analysis.

Item No.	Item	Corrected item—total correlation	Cronbach’s alpha if item deleted	Corrected item—total correlation	Cronbach’s alpha if item deleted
1	When making an appointment in this practice, I had to give the reason for an appointment.	0.153	0.901	Removed	removed
2	The appointment system in this practice is flexible.	0.525	0.745	0.549	0.903
3	During the consultation, team members gave me enough information on self-care.	0.634	0.737	0.682	0.889
4	During the consultation, team members gave me understandable information about my health and planned treatment.	0.642	0.738	0.706	0.888
5	During the consultation, I was able to express my expectations regarding my treatment plan to the team.	0.624	0.738	0.680	0.890
6	In this practice, all patients are treated equally by the whole team.	0.628	0.737	0.692	0.889
7	Each team member in this practice knows their role.	0.650	0.739	0.716	0.887
8	Team members in this practice respect each other.	0.629	0.742	0.698	0.889
9	In this practice, the team handles my data confidentially.	0.615	0.747	0.684	0.891
10	In this practice, all patients are treated with respect by the whole team.	0.628	0.745	0.714	0.888

Therefore, the final version of our Interprofessional Team Approach Evaluation Scale consisted of 9 items.

The mean value of the summary score of the 9-item Interprofessional Team Approach Evaluation Scale was 95.1 ± 9.0 points (on a scale from 0 to 100); the minimum was 33.3 and the maximum 100.0, with a median of 100.0 and a variance of 80.7. The dataset was not normally distributed (Shapiro-Wilk test of normality was 0.62 with p < 0.001). The mean value of the factor Team Approach was 4.8 ± 0.4 (the minimum was 2.6 and maximum 5, on a scale from 1 to 5) and for the factor Person-Centeredness 4.7 ± 0.4 (the minimum was 1.3 and maximum 5.0, on a scale from 1 to 5).

The patients evaluated their team as very high in confidentiality, equity, and respect, and gave the lowest evaluation to the flexibility of the appointment system (Table 5).

**Table 5:** Respondents’ evaluation of the team approach based on the interprofessional team approach evaluation scale.

Item	Mean (standard deviation)	Median (minimum, maximum)
The appointment system in this practice is flexible.	4.7 (0.6)	5.0 (1, 5)
During the consultation, team members gave me enough information on self-care.	4.8 (0.5)	5.0 (1, 5)
During the consultation, team members gave me understandable information about my health and planned treatment.	4.8 (0.5)	5.0 (1, 5)
During the consultation, I was able to express my expectations regarding my treatment plan to the team.	4.8 (0.5)	5.0 (1, 5)
In this practice, all patients are treated equally by the whole team.	4.9 (0.5)	5.0 (1, 5)
Each team member in this practice knows their role.	4.8 (0.5)	5.0 (1, 5)
Team members in this practice respect each other.	4.8 (0.4)	5.0 (1, 5)
In this practice, the team handles my data confidentially.	4.9 (0.4)	5.0 (1, 5)
In this practice, all patients are treated with respect by the whole team.	4.9 (0.4)	5.0 (1, 5)

## Discussion

### Summary of main findings

The Interprofessional Team Approach Evaluation Scale assessing interprofessional team-based care in primary healthcare from the patients’ point of view, which adds a new approach to teamwork measurements and to measurements of patient satisfaction. The results of our study suggest that this scale could be a valid and reliable tool for assessing patient evaluation of family medicine practice teams in Slovenian settings. Team Approach and Person-Centeredness emerged as the key factors of the scale. These factors were also shown to be reliable. The patients evaluated their team as very high in confidentiality, equity, and respect, and gave the lowest evaluation to the flexibility of the appointment system.

#### Comparison to other tools

The Nurse Practitioner Evaluation Scale [9] and the European Patients Evaluation of General Practice Care (EUROPEP) [23] were used in the same settings for patient evaluation of family physicians and nurse practitioners. The EUROPEP scale includes five aspects of care: relation and communication; medical care; information and support; continuity and cooperation; and facilities, availability and accessibility [23]. The Nurse Practitioner Evaluation Scale involves three factors: the Clinical Approach, the Comprehensive Approach, and the Person-centred Approach) [9]. The latter factor was also found in our Interprofessional Team Approach Evaluation Scale.

The General Practice Assessment Questionnaire [18] was developed from the General Practice Assessment Survey [24], and has been found to be a valid and reliable tool for use in general practice [18]. However, it evaluates each member of the family practice team individually, and therefore does not evaluate a team approach to patients. The Primary Care Assessment Tool [19] contains seven factors: first contact accessibility, first contact utilisation, longitudinal interpersonal relationships, comprehensiveness of services available, comprehensiveness of services received, coordination, and community orientation. It also evaluates the patient’s experience mainly with physicians, not with the team as a whole. The Consultation Satisfaction Questionnaire [22] looks at four different areas of satisfaction: general satisfaction, professional care, depth of relationship, and perceived time. Again, it evaluates the physician, not the team. The Nurse Practitioners Satisfaction Survey [20] and the General Practice Nurse Satisfaction Scale [21] evaluate the nurse practitioners working in a family practice team [25–29].

### Factor 1

The Team Approach factor was not recognised in our previously developed tool, which measured patient satisfaction with nurse practitioners within a family medicine team [9].

There are many instruments which measure different aspects of teamwork [6, 7]. Some of them focus in patient safety, such a SafeQuest [25] and a Safety Attitudes Questionnaire [26], some measure team relationships, such as a Survey of Organisational Attributes for Primary Care [27], and some measure working environment such as Clinical Staff Questionnaire [28]. However, only one scale measures interprofessional team-based approach from the viewpoint of the patients but it was developed for the emergency care settings [29]. Our scale, on the other side, offers the measure of the interprofessional teamwork from the aspect of patients [25, 26, 30].

### Factor 2

A Person-Centeredness factor was already recognised in our previously developed tool, which measured patient satisfaction with nurse practitioners within a family medicine team as a patient-centred approach [9]. Both concepts differ in terms of the object of care; a patient is the one who is sick or being treated, and a person is a human being [3], but both concepts share the same approach. We decided to use a broader concept—Person-Centeredness—as within family medicine not all people that come for a consultation correspond to the term patient. Person-Centeredness is an important part of the family medicine approach and also has an important role in quality assurance [31]. It has also been recognised in other instruments that measure teamwork or patient satisfaction [19, 23]. Person-Centred care cannot be practiced by just one member of a team but only by the whole team, so this factor is important for teamwork, and is obviously also recognised by the patients.

### Descriptive results of the study

The patients assessed the team approach in family medicine practices very highly. This is also in line with the previous studies on patient satisfaction in Slovenian family medicine practices [9, 32]. The item that received least points was the flexibility of the appointment system. This problem seems to characterise Slovenian family medicine practices already for some time as other studies showed [9, 32, 33].

### Implications of the study

The results of our study showed two factors that might be important in the evaluation by patients of the teamwork approach in family medicine. Still, other factors could also be important within this context, as shown by other studies focusing on teamwork [6]. Our study pointed out the need to develop a comprehensive theoretical framework of family medicine team competencies, which could then be used to develop a comprehensive tool for evaluating the team approach to patients. Several comprehensive frameworks for family medicine already exist, but they all focus only on one member of the team [34–37]. A competency framework for the whole team is needed, as some studies have shown that the competencies of individual members of the team are not fully understood and could be a source of less effective teamwork [21, 38, 39].

Future studies should focus on developing the aforementioned framework and consequently a comprehensive tool for its measurement.

### Strengths and limitations of the study

This was the first study that dealt with patient evaluations of family medicine teams in Slovenia and probably also internationally. We developed a new tool for assessing patient evaluation of the team approach in family medicine practices through a multistep process ensuring its validity and reliability. We used consensus development panels which are a qualitative method for obtaining agreement in areas of uncertainty or where there is a lack of definitive information. Furthermore, consensus development panels help bring professionals together to directly comment and develop tools and techniques [34]. The development process could have been more rigorous, involving focus groups with experts including representatives of patients which would result in a more comprehensive framework. This could also be a reason why some factors recognised in other studies were not recognised in our study. Also, the content validity could be confirmed by the content validity index which we did not calculate but used qualitative information on items’ content validity as judged by the experts. We also did not perform a collection of a response process and we did not split the respondents to two groups to determine the coherency of the concepts with exploratory and factor analyses. These, too, could be considered as limitations of our study. The strength of this study is the large sample of patients included in the quantitative part of the study, which means the results can be generalised.

## Conclusion

This study provided a new scale for the evaluation of patient satisfaction with the family medicine team as a whole. It opened the question of family medicine team competencies, and pointed towards the need to develop a family medicine team competency framework and a comprehensive tool for its measurement. This would strengthen the team approach and possibly enhance the safety culture in family medicine.

## Supporting information

S1 Dataset Dataset. This supporting information contains the anonymised dataset used in the study.
(XLSX)
